# Electroacupuncture Attenuates 5′-Guanidinonaltrindole-Evoked Scratching and Spinal c-Fos Expression in the Mouse

**DOI:** 10.1155/2013/319124

**Published:** 2013-06-25

**Authors:** Yi-Hung Chen, Han-Yin Yang, Chia-Hsien Lin, Nae J. Dun, Jaung-Geng Lin

**Affiliations:** ^1^Graduate Institute of Acupuncture Science, China Medical University, Taichung 40402, Taiwan; ^2^School of Chinese Medicine, China Medical University, Taichung 40402, Taiwan; ^3^Department of Health Industry Management, Kainan University, Taoyuan 33857, Taiwan; ^4^Department of Pharmacology, Temple University School of Medicine, Philadelphia, PA 19140, USA

## Abstract

The present study was undertaken to investigate the influence of electroacupuncture (EA) on compulsive scratching in mice and c-Fos expression elicited by subcutaneous (s.c.) administration of a known puritogen, 5′-guanidinonaltrindole (GNTI) to the neck. Application of EA to Hegu (LI4) and Quchi (LI11) acupoints at 2 Hz, but not 100 Hz, attenuated GNTI-evoked scratching. In mice pretreated with the *µ* opioid receptor antagonist naloxone, EA 2 Hz did not attenuate GNTI-evoked scratching, whereas EA at 2 Hz did attenuate GNTI-evoked scratching in mice pretreated with the **κ** opioid receptor antagonist nor-binaltorphimine. Moreover, intradermal (i.d.) administration of the selective *µ* opioid receptor agonist [d-Ala2, N-Me-Phe4, Gly5-ol]-enkephalin acetate (DAMGO) attenuated GNTI-evoked scratching behavior, while s.c. administration of DAMGO was ineffective. GNTI provoked c-Fos expression on the lateral side of the superficial layer of the dorsal horn of the cervical spinal cord. Application of 2 Hz EA to LI4 and LI11 decreased the number of c-Fos positive nuclei induced by GNTI. It may be concluded that application of 2 Hz EA to LI4 and LI11 attenuates scratching behavior induced by GNTI in mice and that the peripheral *µ* opioid system is involved, at least in part, in the anti-pruritic effects of EA.

## 1. Introduction

Itch is a sensation that provokes the desire or reflex to scratch [1, 2]. It is a leading sign of skin diseases but also occurs in some systemic diseases. Yosipovitch et al. [3] classified itch according to its origin: (1) pruritoceptive (originating in the skin, due to inflammation, dryness, or other skin damage); (2) neuropathic (arising out of disease located at any point along the afferent pathway); (3) neurogenic (central in origin, but without evidence of neuropathology); and (4) psychogenic, as in the delusional state of parasitophobia. Recent advances in pruritus research have partially elucidated the signaling molecules and neuronal pathways involved in itch transmission [4]. Pruritus may cause considerable debilitation, but treatment is often ineffective [5]. Antihistamines may relieve itching in various allergies, but they do not lessen itch in skin, liver, or kidney diseases.

Acupuncture has been used in traditional Chinese medicine for over 2,500 years [6]. Practice of acupuncture as a treatment modality has steadily increased in Western countries [7] and is now known as “complementary medicine” due to its efficacy in the treatment of certain physical and psychological disorders. In 1997, the US National Institutes of Health issued a report in which acupuncture is listed as an effective treatment modality against many disorders with fewer side effects as compared to medications or surgery [7]. The WHO has published a guide listing 64 disorders/symptoms, including clinical conditions associated with pruritus, where acupuncture treatment can improve or ameliorate the severity of clinical conditions in question [8]. Two different strategies are employed in acupuncture therapy, that is, manual acupuncture (MA) and electroacupuncture (EA); the latter is a modified form of traditional MA. Most studies use EA because it can be standardized according to frequency, voltage, waveform, length, and so forth.

Several clinical studies have demonstrated the efficacy of acupuncture in alleviating itch. In particular, acupuncture has been shown to relieve histamine-induced itch [9], type I hypersensitivity itch [10], nasal itch [11], refractory uremic pruritus [12], and neurogenic pruritus [13]. In these studies, the Hegu (LI4) and Quchi (LI11) acupoints are most commonly used. The mechanism whereby acupuncture relieves itch has not been studied in detail.

The highly selective *κ* opioid receptor antagonist 5′-guanidinonaltrindole (GNTI) [14] has been shown to precipitate immediate, excessive scratching in mice [15], using the itch model described by Kuraishi et al. [16]. The scratch behavior induced by subcutaneous (s.c.) injection of GNTI to the neck is abolished by the *κ* opioid agonist, nalfurafine [15]. Expression of the *c-fos* gene or Fos protein is widely used as a marker in identifying the neural pathways involved in the integration of noxious inputs [17–21]. Nojima et al. [22] reported spontaneous itch-related scratching behavior, which can be correlated with c-Fos expression in the superficial dorsal horn of the spinal cord in a rat model of dry skin. Inan et al. [23] reported that GNTI provoked c-fos expression in neurons located at the lateral area of the dorsal horn. 

In this study, EA experiments were conducted to (1) establish the applicability of EA in alleviating GNTI-induced scratch in a murine model; (2) determine the frequency-dependent anti-scratch activity of EA; (3) correlate the efficacy of EA with expression of Fos protein in the spinal cord; and (4) define the involvement of opioid receptors in EA-mediated anti-scratch activity.

## 2. Methods

### 2.1. Animals

Male ICR mice (25–30 g; BioLasco Taiwan Co., Ltd., Taiwan) were housed under a 12 : 12 light/dark cycle with food and water available *ad libitum *in our animal facility for at least 4 days prior to the experiments, which were conducted between 10:00 and 17:00. The experimental procedures were approved by the China Medical University Institutional Animal Care and Use Committee, in accordance with the care and use of laboratory animal guidebook from the Chinese Taipei Society of Laboratory Animal Sciences. The experiments were designed to keep the number of mice at a minimum and care was taken to minimize suffering.

### 2.2. Electroacupuncture

**Figure 1:**
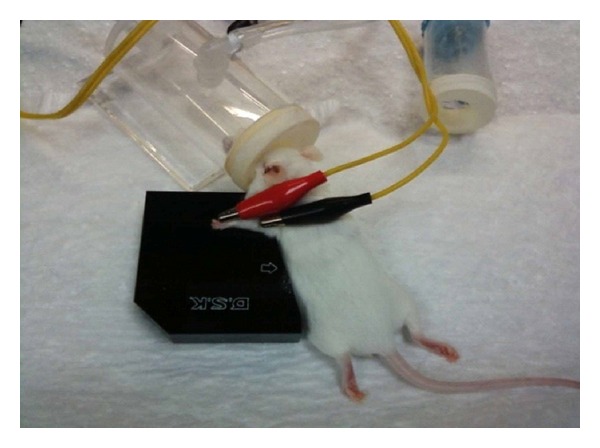
Photograph of a mouse under isoflurane anesthesia with acupuncture needles inserted at Hegu (LI4) and Quchi (LI11) acupoints. LI4 is located on the first dorsal interossei, radial to the midpoint of the second metacarpal bone in the forelimb. LI11 is located in the depression on the lateral end of the cubital crease in the forelimb, when the elbow is fully flexed [24].

Mice were individually acclimated in rectangular observation boxes for 1 hr and then anesthetized with 1.5% isoflurane. Under anesthesia, a pair of stainless steel acupuncture needles (36-gauge) was inserted 2 mm deep into the murine equivalent of the human Hegu (LI4) and Quchi (LI11) acupoints. The murine LI4 is located on the first dorsal interossei, radial to the midpoint of the second metacarpal bone in the forelimb. The murine LI11 is located in the depression on the lateral end of the cubital crease in the forelimb, when the elbow is fully flexed [24]. A pair of acupuncture needles (32-gauge) was also inserted 3-4 mm deep into the bilateral murine equivalent of the human Zusanli (ST36). The ST36 is located on the anterolateral side of the hindlimb near the anterior crest of the tibia, in a depression below the knee on the tibialis anterior muscle [26, 27]. EA stimuli were delivered by an EA Trio 300 stimulator (Ito, Japan) at an intensity of 2 mA for 20 min at either 2 or 100 Hz with a pulse width of 150 *μ*s [25].  Figure 1 shows an anesthetized mouse receiving EA applied to LI4 and LI11.

### 2.3. Effect of Electroacupuncture on Pruritogen-Evoked Scratching Behavior

After termination of EA, mice were allowed to recover from anesthesia for 30 min. Saline or GNTI (0.3 or 0.6 mg/kg) were injected s.c. in the midline behind the neck. To minimize the effects of anesthesia, the number of hind leg scratches directed to the back of the neck was counted for up to 40 min [19, 23, 25, 28–30]. All test agents were administered in a dose volume of 0.01 mL/g.

### 2.4. Opioid Receptor Antagonist Study

The *μ* opioid receptor antagonist, naloxone (s.c.; 1 mg/kg) [30, 31], and the *κ* opioid receptor antagonist, nor-binaltorphimine (s.c.; 10 mg/kg) [25], were used to pretreat mice. Naloxone (s.c.; 1 mg/kg) was administered at 70 min before GNTI injection; nor-binaltorphimine (s.c.; 10 mg/kg) was administered at 24 hr before GNTI injection [25]. EA was applied to the LI4 and LI11 acupoints before GNTI administration. After termination of EA, mice were allowed to recover from anesthesia for 30 min. GNTI (0.3 mg/kg) was injected s.c. in the midline behind the neck. The number of scratches was counted for 40 min after pruritogen injection.

### 2.5. Opioid Receptor Agonist Study

This study used the *μ* opioid receptor agonist [d-Ala2, N-Me-Phe4, Gly5-ol]-enkephalin acetate (DAMGO) [32]. Mice were individually acclimated in rectangular observation boxes for at least 2 h and then injected intradermally (i.d.) or s.c. with DAMGO (10 nmol/site [33]) at the back of the neck. Ten minutes later, the pruritogen GNTI (0.3 mg/kg) was injected s.c. in the midline behind the neck of mice. The number of hind leg scratches directed to the back of the neck was counted for 30 min [30, 34]. 

### 2.6. Fos Immunoreactivity in Cervical Spinal Cords

The immunohistochemistry procedure was similar to that described by Inan et al. [25]. In brief, 2 hr after the injection of saline or GNTI (0.6 mg/kg), animals were deeply anesthetized with urethane (1.2 g/kg; i.p.) and perfused intracardially with ice-cold 0.1 M phosphate-buffered saline (PBS) followed by 4% paraformaldehyde in 0.1 M PBS. The cervical spinal cords were removed and postfixed in the same fixative overnight at 4°C. Tissue samples were transferred to 30% sucrose solution for at least 3-4 d before sectioning. Cervical spinal cord (C5–C7) sections (30 *μ*m) were cut at −25°C with a freezing microtome (Leica CM3050 S). Free-floating sections were stored in PBS at 4°C until immunohistochemistry was performed. Fifteen cervical spinal cord sections from each mouse were randomly selected for immunohistochemistry.

Tissues were processed for c-Fos immunoreactivity by the avidin-biotin complex procedure [35]. They were first treated with 3% H_2_O_2_ to quench endogenous peroxidase activity, washed twice for 10 min with PBS, and blocked with 20% normal goat serum (1 : 20) at room temperature for 2 hr. The sections were then incubated in a shaker for 2 days at 4°C with rabbit c-Fos antibody (1 : 1000 dilution) (sc-52; Santa Cruz Biotechnology, Santa Cruz, CA, USA). After thorough rinsing, sections were incubated in biotinylated anti-rabbit immunoglobulin G secondary antibody (1 : 300 dilution; Vector Laboratories, Burlingame, CA, USA) for 2 hr at room temperature. Following two 10 min rinses with PBS, the sections were incubated in an avidin-biotin-peroxidase complex at room temperature for 90 min (1 : 300 dilution; Vectastain ABC Elite kit, Vector Laboratories). Following three 10 min washes in Tris-buffered saline, sections were reacted with 0.05% diaminobenzidine/0.001% H_2_O_2_ solution for 4-5 min and washed 3 times for 10 min with Tris-buffered saline. Sections were mounted on slides with 0.25% gel alcohol, air-dried, dehydrated with graded ethanol (50%, 70%, 95%, and 100%, for 6 min each) followed by xylene (twice for 10 min), and coverslipped with Permount. c-Fos positive nuclei were observed under a light microscope and counted at 200x magnification.

### 2.7. Chemicals

Diaminobenzidine was purchased from Calbiochem (La Jolla, CA, USA); GNTI, DAMGO, naloxone hydrochloride, and nor-binaltorphimine dihydrochloride were purchased from Sigma Chemical Co. (St. Louis, MO, USA). 

### 2.8. Data Analysis

Statistical comparisons were performed with Student's *t*-test or one-way ANOVA followed by Tukey's post hoc test. All data are expressed as the mean ± standard error of the mean (S.E.M.). *P* < 0.05 was considered statistically significant.

## 3. Results

### 3.1. Attenuation of GNTI-Evoked Scratching by EA at 2 Hz but Not 100 Hz

**Figure 2:**
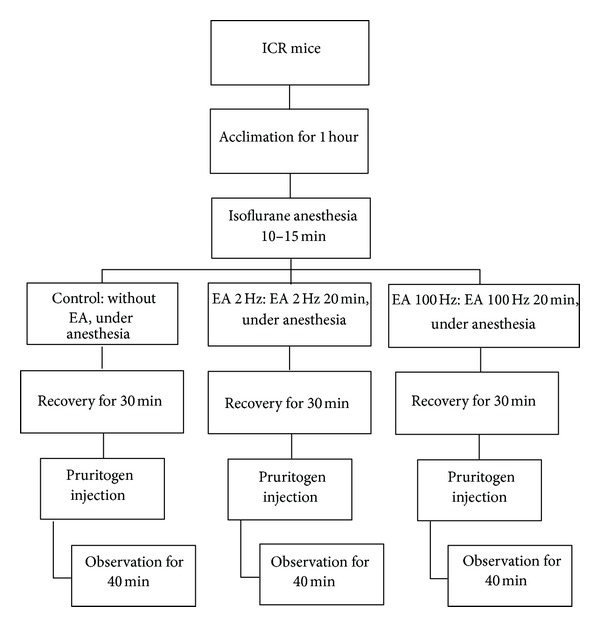
Flow chart depicting steps involved in the EA study.

**Figure 3:**
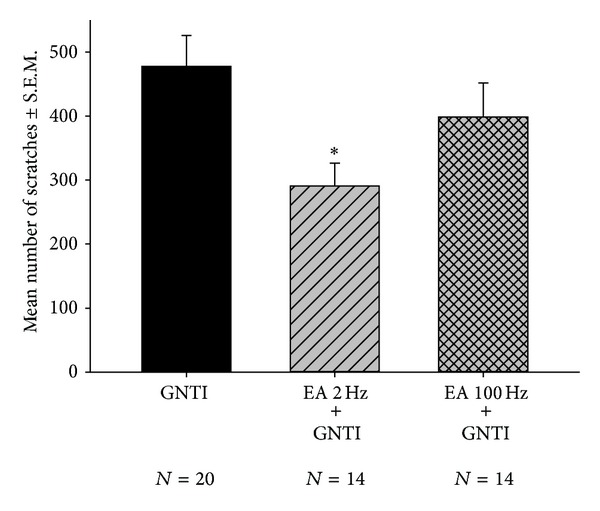
Effects of EA at 2 Hz and 100 Hz on GNTI- (0.3 mg/kg) induced scratching. EA was applied to the LI4 and LI11 acupoints before GNTI administration. The number of scratches was counted for 40 min after pruritogen injection. EA at 2 Hz, but not 100 Hz, attenuated GNTI-induced scratching behavior (*P < 0.05 compared to controls). Between-group comparisons for each group were performed by ANOVA, followed by Tukey's test.

To test the effects of EA at two different frequencies (2 and 100 Hz) applied to LI11 and LI4 on GNTI-induced scratching behavior, mice were divided into 3 groups: (1) control, (2) 2 Hz EA, and (3) 100 Hz EA (Figure 2). In contrast to the EA groups, mice in the control group received isoflurane but did not undergo EA. After anesthesia, mice in each group were allowed to recover for 30 min and then injected with GNTI (0.3 mg/kg). Mice started scratching 2-3 min following the GNTI injection. This strong, stereotypic behavior continued throughout the 40 min observation period. At the end of 40 min, the averaged numbers of scratches in the control, 2 Hz EA, and 100 Hz EA groups were 477.7 ± 48 (*n* = 20), 290.6 ± 36.1 (*n* = 14), and 398.3 ± 53.7 (*n* = 14), respectively (Figure 3). Compared with the control group, pretreatment with 2 Hz EA decreased GNTI- (0.3 mg/kg) evoked scratching by 39.2% (*P* < 0.05), whereas 100 Hz EA pretreatment had no such effect (*P* > 0.05).

### 3.2. EA at 2 Hz Applied to ST36 Did Not Affect GNTI-Induced Scratching

**Figure 4:**
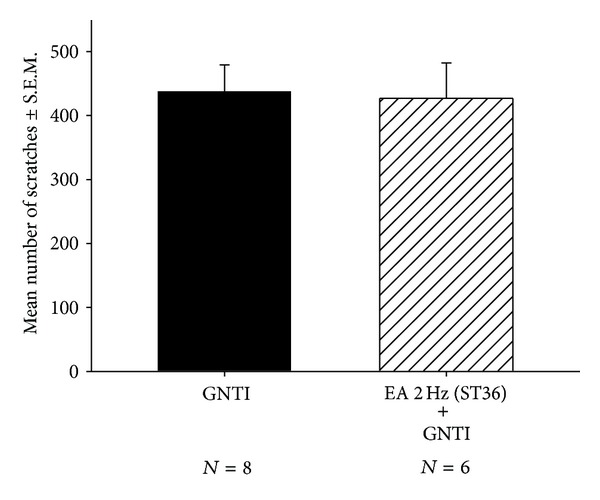
Effects of EA at 2 Hz on GNTI- (0.3 mg/kg) induced scratching. EA was applied to the bilateral ST36 acupoints prior to GNTI administration. The number of scratches was counted for 40 min after GNTI injection. EA at 2 Hz had no effect upon GNTI-induced scratching behavior. Between-group comparisons for each group were performed by Student's *t*-test.

To test the effects of EA at 2 Hz applied to bilateral ST36 acupoints on GNTI-induced scratching behavior, mice were divided into 2 groups: (1) control, (2) 2 Hz EA applied to bilateral ST36 (Figure 4). At the end of a 40 min observation period, the averaged numbers of scratches in the control group and 2 Hz EA were 438.1 ± 41.1 (*n* = 8) and 427.2 ± 55.4 (*n* = 6), respectively (Figure 4). The between-group number of scratches did not differ significantly. It appears that pretreatment with 2 Hz EA to bilateral ST36 did not affect GNTI- (0.3 mg/kg) evoked scratching.

### 3.3. Naloxone Prevented the Effects of EA (2 Hz) on GNTI-Induced Scratching

**Figure 5:**
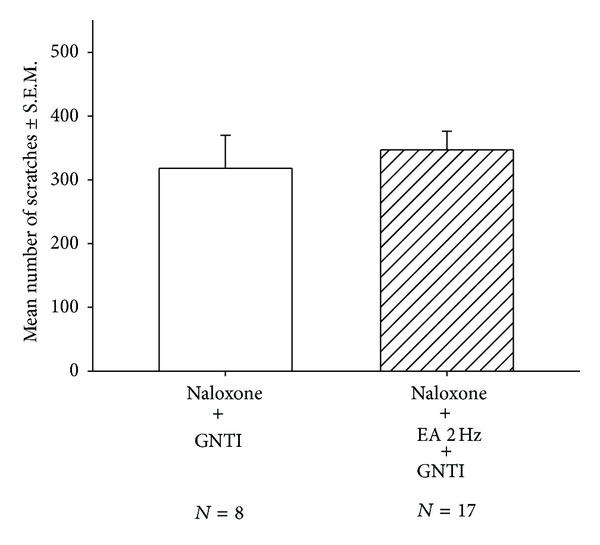
Effects of EA at 2 Hz on GNTI- (0.3 mg/kg) induced scratching in naloxone-pretreated mice. Naloxone (s.c.; 1 mg/kg) was administered 70 min before GNTI injection, that is, 5 min before isoflurane anesthesia for EA. EA was applied to the LI4 and LI11 acupoints before GNTI administration. The number of scratches was counted for a period of 40 min after pruritogen injection. EA failed to attenuate GNTI-induced scratching behavior in naloxone-pretreated mice. Between-group comparisons for each group were performed by Student's *t*-test.

To test whether EA attenuates GNTI-induced scratching in mice pretreated with naloxone (1 mg/kg; s.c.), animals were divided into 2 groups: (1) naloxone-pretreated controls and (2) naloxone-pretreated plus EA. The scratching behavior was observed for 40 min. After 40 min, the averaged numbers of scratches in the naloxone-pretreated controls and naloxone-pretreated plus EA mice were 318.4 ± 51.6 (*n* = 8) and 347.2 ± 28.8 (*n* = 17), respectively (Figure 5). EA did not significantly attenuate GNTI-induced scratching behavior in naloxone-pretreated mice.

### 3.4. Nor-Binaltorphimine Did Not Alter the Effects of EA (2 Hz) on GNTI-Induced Scratching

**Figure 6:**
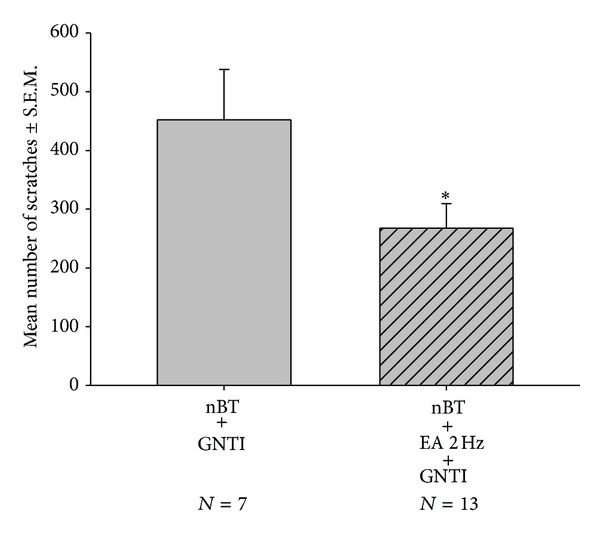
Effects of EA at 2 Hz on GNTI- (0.3 mg/kg) induced scratching in nor-binaltorphimine-pretreated mice. Nor-binaltorphimine (10 mg/kg) was subcutaneously administered 24 hr before GNTI injection [25]. EA was applied to the LI4 and LI11 acupoints before GNTI administration. The number of scratches was counted for 40 min after pruritogen injection. EA attenuated GNTI-induced scratching behavior in nor-binaltorphimine-pretreated mice (*P < 0.05). Between-group comparisons for each group were performed by Student's *t*-test.

To test whether EA attenuates GNTI-induced scratching in mice pretreated with nor-binaltorphimine (10 mg/kg; s.c.), animals were divided into 2 groups: (1) nor-binaltorphimine-pretreated controls and (2) nor-binaltorphimine-pretreated plus EA. The scratching behavior was observed for 40 min. After 40 min, the averaged numbers of scratches in the nor-binaltorphimine-pretreated controls and (2) nor-binaltorphimine plus EA mice were 452.3 ± 85.9 (*n* = 7) and 267.5 ± 41.9 (*n* = 13), respectively (Figure 6). Thus, EA significantly attenuated GNTI-induced scratching behavior by 41% in nor-binaltorphimine-pretreated mice.

### 3.5. Attenuation of GNTI-Induced Scratching by Intradermal Administration of DAMGO, Not by Subcutaneous Administration of DAMGO

**Figure 7:**
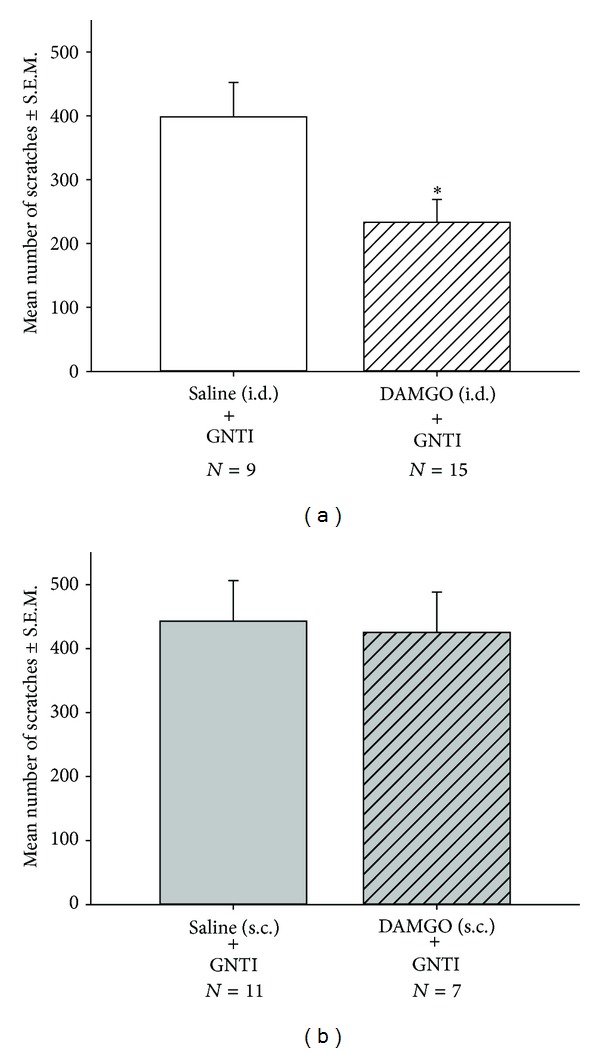
Effects of DAMGO by i.d. injection (a) and by s.c. injection (b) on GNTI- (0.3 mg/kg) induced scratching. (a) Mice were administered an i.d. injection of saline or DAMGO (10 nmol) at 15 min before GNTI (0.3 mg/kg). (b) Mice were administered a s.c. injection of saline or DAMGO (10 nmol) at 15 min before GNTI (0.3 mg/kg). The number of scratches was counted for 30 min after GNTI injection. DAMGO (10 nmol/site) attenuated GNTI-induced scratch behavior by i.d. administration but not s.c. administration. (*P < 0.05 compared to controls). Between-group comparisons for each group were performed by Student's *t*-test.

To further clarify the roles of peripheral versus central *μ* opioid receptors in the anti-pruritic effect of EA, we compared the effects of DAMGO by i.d. and s.c. injection. In the intradermal studies, mice received injections of saline (controls) or DAMGO (10 nmol/site) at the back of the neck. After 15 min, animals were injected with GNTI (0.3 mg/kg; behind the neck). After 30 min, the number of scratches was 398.3 ± 53.5 (*n* = 9) for the control group and 233.1 ± 35.6 (*n* = 15) for the DAMGO-treated group (Figure 7(a)). Thus, intradermal pretreatment with DAMGO significantly decreased the number of scratches induced by GNTI (0.3 mg/kg) by 41.5% (*P* < 0.05) as compared to the control group. 

In the subcutaneous studies, similar procedures were performed as above, while DAMGO was injected s.c. at the back of the neck. After 30 min, the number of scratches for the control group was 442.6 ± 63.4 (*n* = 11) compared with 425.3 ± 62.8 (*n* = 7) for the s.c. DAMGO-treated group (Figure 7(b)). Thus, subcutaneous pretreatment with DAMGO did not affect scratch behavior induced by GNTI (0.3 mg/kg).

### 3.6. 2 Hz EA Attenuates GNTI-Induced Scratching at a Higher Dose

To evaluate the effects of 2 Hz EA on a higher dose of GNTI-induced scratching behavior, the mice were divided into 2 groups: (1) GNTI 0.6 mg/kg (controls) and (2) GNTI 0.6 mg/kg with 2 Hz EA. After 40 min, the averaged numbers of scratches for the control and 2 Hz EA groups were 618.2 ± 78.7 (*n* = 10) and 383.1 ± 62 (*n* = 10) (Figure 11(a)). Pretreatment with 2 Hz EA significantly decreased GNTI- (0.6 mg/kg) induced scratching by 38% as compared to the control group (*P* < 0.05).

### 3.7. Attenuation of GNTI-Induced c-Fos Expression by EA

**Figure 8:**
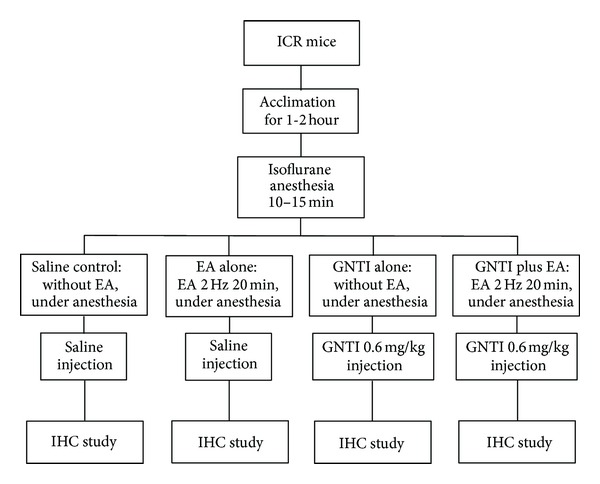
Flow chart depicting steps involved in the immunohistochemistry (IHC) study.

This series of studies was undertaken to test the effects of EA on GNTI- (0.6 mg/kg) induced c-Fos expression in superficial layers of the cervical spinal cord. Mice were divided into 4 groups: saline control; EA alone; GNTI alone; GNTI plus EA (Figure 8). 

**Figure 9:**
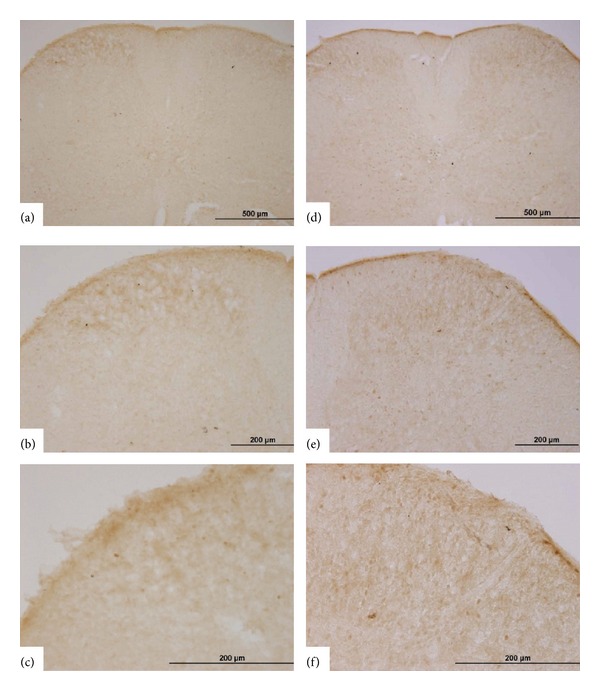
Representative photomicrographs of c-Fos expression in the spinal cord. c-Fos was not detected in the superficial layers of the dorsal horn following saline injection (a)–(c) and after EA (2 Hz) application at the LI4 and LI11 acupoints (d)–(f). (a)–(c) represent one spinal section, with increasing magnification, while (d)–(f) are from another spinal section, with increasing magnification.

**Figure 10:**
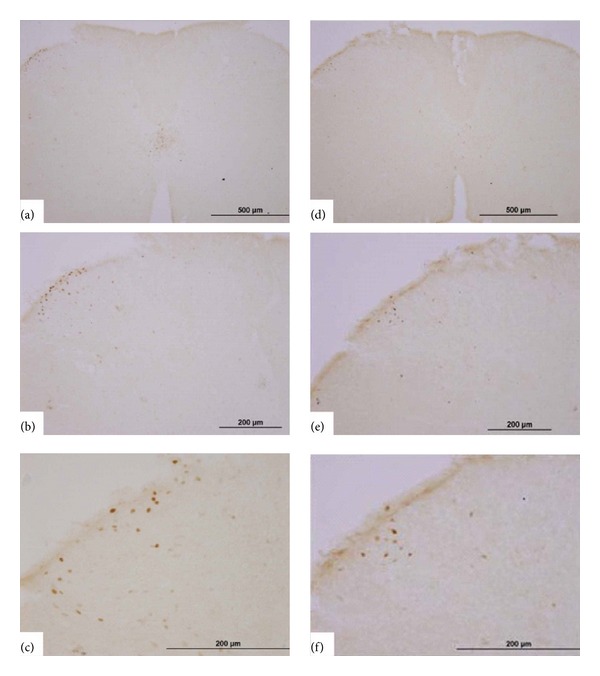
Representative photomicrographs of c-Fos expression induced by GNTI in the spinal cord and the effects of EA. GNTI (0.6 mg/kg; s.c.; behind the neck) induced c-Fos expression on the lateral side of the superficial lamina of the dorsal horn of the cervical spinal cord without (a)–(c) and with (d)–(f) EA (2 Hz) pretreatment. (a)–(c) represent one spinal section, with increasing magnification, while (d)–(f) are from another spinal section, with increasing magnification.

**Figure 11:**
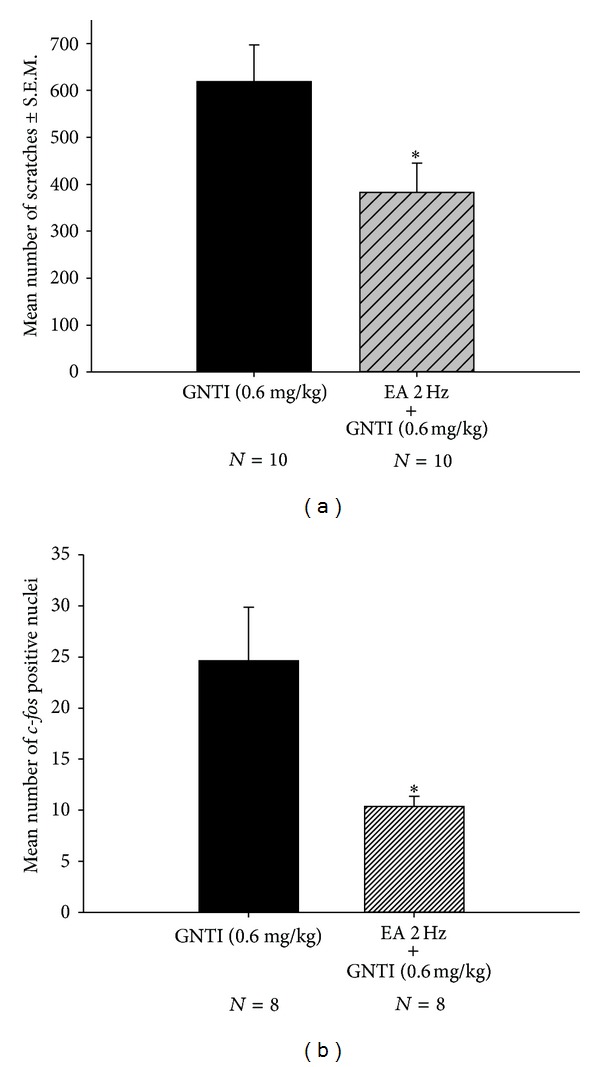
Effects of EA on GNTI- (0.6 mg/kg) induced scratching behavior (a) and c-Fos expression (b). (a) Effects of EA 2 Hz (applied to the LI4 and LI11 acupoints) on GNTI- (0.6 mg/kg) induced scratches. The number of scratches was counted for 40 min after injection of GNTI. EA at 2 Hz reduced GNTI- (0.6 mg/kg) induced scratching behavior (*P < 0.05 compared to control by Student's *t*-test). (b) The numbers of c-Fos positive nuclei were counted and averaged from 12 randomly chosen cervical sections from each animal in each group. EA at 2 Hz was applied to the LI4 and LI11 acupoints before administration of GNTI (0.6 mg/kg, s.c.). EA (2 Hz) significantly decreased the number of c-Fos positive nuclei (*P < 0.05, Student's *t*-test; *N*: the number of animals).

GNTI significantly increased the number of c-Fos positive nuclei on the lateral side of the superficial layers of the dorsal horn as compared to the saline control group (*P* < 0.01; Figures 9 and 10). While 2 Hz EA alone had no significant effect on c-Fos expression (Figure 9), pretreatment with 2 Hz EA significantly reduced the number of c-Fos positive nuclei induced by GNTI (Figures 10 and 11(b); *P* < 0.05; *n* = 8). 

## 4. Discussion

### 4.1. EA Inhibits Pruritogen-Induced Scratching

While the influence of acupuncture on pain has been extensively studied, much less is known about the effects of acupuncture on itch. Several clinical studies have demonstrated the efficacy of acupuncture on itch in humans [12, 36–38]. Investigations by Han et al. [25] explored the anti-pruritic effects of acupuncture in a rat model of hindlimb scratching. In their study, itch-associated scratching behavior was induced by i.d. injections of 5-HT into the rostral back, and MA or EA at the frequency of 2 and 120 Hz significantly reduced the number of scratching bouts over a period of 60 min as compared to that of control rats. Further, pretreatment of the rats with nor-binaltorphimine inhibited the anti-pruritic effect of 120 Hz EA. The authors suggest that the anti-pruritic effects of acupuncture using high-frequency EA is due to *κ* opioid receptor activation.

In the present study, we first established a different animal scratching model, that is, GNTI-induced scratching behaviors [23] suitable for assessing the antipruritic effects of EA. Our results show that application of 2 Hz EA to LI4 and LI11 significantly diminished GNTI-induced scratching behavior, whereas 100 Hz EA had no such effect. EA (2 Hz) applied to ST36 had no significant effect upon GNTI-induced scratching behavior. To the best of our knowledge, this is the first report to demonstrate that EA inhibits scratching in a frequency-dependent manner by a pruritogen other than 5-HT [25]. The findings indicate that different frequencies of EA should be considered in the treatment of pruritus caused by different pruritogens. Further, LI4 and L11 are preferred over the ST36 for pruritus treatment, in spite of ST36 being recognized as the important acupoint for acupuncture analgesia [39–41]. 

### 4.2. Pain and Itch

The sensations of pain and itch share many interactions in acute transmission and sensitization processes, but they have differences as well. Pain sensation evokes withdrawal behavior, while itch evokes scratching as a reflex or conscious mechanical stimulation to relieve the hazardous stimulant and act as a helpful warning of potential hazards [42].

Separate sets of neurons mediate itch and pain inputs from the peripheral nerve [43]. Primary afferents for pain sensation are mechanosensitive and mechanoinsensitive A*δ*- and polymodal C-fibers [44, 45], whereas primary afferents for itch sensation are histamine-activated mechanoinsensitive C-fibers [46] and cowhage-activated mechano sensitive fibers [47]. Although stimuli follow the spinothalamic pathway, neurons stimulated by histamine project to the posterior part of the ventromedial nucleus of the thalamus [45], whereas neurons stimulated by pain project to the ventroposterior lateral nucleus of the thalamus [48]. 

Recent research has identified that, rather than there being a clear set of separate specific mediators for pain and itch, most receptor systems may participate in both pain and itch processing in the keratinocyte and nerve ending [42]. Substance P, interleukins, opioids, endothelins, proteases, and others may be involved in itch and pain sensations [42]. However, recent research has proposed that gastrin-releasing peptide, abbreviated as GRP, is a key neurotransmitter in itch sensation in the spinal cord [49, 50]. 

Itch may be inversely related to pain [51]. Evidence indicates the involvement of the central *μ* opioid receptor system in itch generation. This phenomenon is particularly relevant to spinally administered *μ* opioid receptor agonists, which induce segmental analgesia but also induce itch [52]. Intracerebroventricular administration of *β*-funaltrexamine, a selective *μ* opioid receptor antagonist, inhibited the scratching behavior induced by intradermal substance P [53]. However, *κ* opioid agonists reduce experimental itch, instead of inducing itch [54], and *κ* opioid antagonists induce itch in animal experiments [55]. While the effects of opioids in the central nervous system have been investigated in detail, local production of opioids in the skin is a recent finding. The role of peripheral opioids on itch remains to be explored.

### 4.3. Peripheral *μ* Opioid Receptors May Be Involved in the Effects of EA

One of the better characterized mechanisms that may underlie EA relates to a release of endogenous opioids and activation of opioid receptors. Early studies demonstrated the role that endogenous opioids may play in acupuncture-induced analgesia [56]. EA at a lower frequency (2 Hz) stimulates the release of *β*-endorphin, enkephalin, and endomorphin within the CNS [57–59], whereas EA at a higher frequency (100 Hz) induces the release of dynorphin [60]. Accordingly, 2 Hz EA analgesia may be mediated by *μ* and *δ* opioid receptors, while 100 Hz EA analgesia is mediated by *κ* opioid receptors. 

To clarify the relationship between the anti-pruritic effect induced by EA (2 Hz) and opioid receptors, we tested the effects of naloxone and nor-binaltorphimine. In mice pretreated with naloxone, a *μ* opioid receptor antagonist, EA 2 Hz did not attenuate GNTI-evoked scratching, whereas when mice were pretreated with nor-binaltorphimine, a *κ* opioid receptor antagonist, EA 2 Hz did successfully attenuate GNTI-evoked scratching. These results reveal that the antipruritic effects of EA (2 Hz) are prevented by a *μ* opioid receptor antagonist. The effect of EA on GNTI-induced scratching may be related to *μ*, but not *κ*, opioid receptors.

As mentioned above, evidence indicates the central *μ* opioid receptor may elicit itch. However, the peripheral *μ* opioid receptor may have a different role in itch sensation [61]. To further clarify the roles of peripheral and central *μ* opioid receptors in the anti-pruritic effects of EA, we compared the effects of DAMGO by i.d. and s.c. injection. It is thought that intradermal injection of DAMGO can locally activate peripheral mu-opioid receptors, while s.c. injection of DAMGO can activate both peripheral and central *μ* opioid receptors. We found that DAMGO (10 nmol/site) attenuated GNTI-induced scratch behavior by i.d. administration but not s.c. administration. The results suggest that only peripheral activation of *μ*-opioid receptors can inhibit GNTI-induced scratching behavior. Similar results were also observed from another study in that i.d. administration of DAMGO reduced endothelin-1 induced-pruritic behavior [61]. It is also noted that EA is able to activate peripheral *μ* receptors on peripheral nerve terminals [62]. These results suggest that the peripheral *μ* opioid receptor may be involved in the anti-pruritic effects of low-frequency EA against GNTI-induced scratching.

### 4.4. EA Decreases GNTI-Induced c-Fos Expression

As mentioned earlier, peripheral receptors uniquely sensitive to pruritogens have yet to be firmly identified. The selectivity of the stimulus appears to be related to a differential connectivity at the spinal level, where a specific class of responsive dorsal horn neurons is located. From there, the stimulus travels via the lateral spinothalamic tract to the thalamus and to the cerebral cortex, where it causes the sensation of itch [46]. The expression of Fos protein or *c-fos* transcript indicates a population of neurons are activated or excited by nociceptive (noxious) inputs [18, 63]. Nojima et al. [22] suggested that spontaneous itch-related scratching behavior is associated with c-Fos expression in the superficial dorsal horn activated by pruritogens including opiates, 5-HT, and histamine. Viewed in this context, the localization of Fos in the dorsal horn may provide a useful indicator relative to the distribution of individual neurons activated by noxious inputs in awake, behaving animals [17, 18, 20–22]. In regard to the area of c-Fos expression in the spinal cord, it is reported that formalin, the pain inducer, induces an increase in c-Fos expression on the medial side of the superficial and deeper layers of the dorsal horn of the lumbar spinal cord in mice, while the pruritogen GNTI induces an increase in c-Fos expression on the lateral side of the superficial lamina of the dorsal horn of the cervical spinal cord [64]. 

In this study, immunohistochemistry was employed to address the issue as to whether a reduction of GNTI-induced c-Fos expression is correlated with EA. The results showed that EA alone did not increase c-Fos expression and that GNTI (0.6 mg/kg) induced c-Fos expression on the lateral side of the superficial layer of the dorsal horn in the cervical cord. Administration of 2 Hz EA to the LI4 and LI11 acupoints decreased the number of c-Fos positive nuclei induced by GNTI. These results further support the contention that EA attenuates noxious stimuli imposed by GNTI. This is the first report demonstrating that EA decreases pruritogen-induced c-Fos expression in a mouse model. 

Several clinical trials have shown that acupuncture or EA relieves histamine-induced itch [9], type I hypersensitivity itch [10], nasal itch [11], refractory uremic pruritus [12], and neurogenic pruritus [13]. Our findings not only support the suitability of EA in relieving pruritus but also underscore the observation that EA should be evaluated at different frequencies to take into account the multiple signaling molecules and mechanisms that may underlie pruritus. 

## 5. Conclusion

EA diminishes the scratching behavior induced by GNTI in a murine model. The peripheral *μ* opioid system is involved, at least in part, in the anti-pruritic effects of EA. Collectively, the application of EA to the LI4 (Hegu) and LI11 (Quchi) acupoints holds promise as an effective anti-pruritic treatment modality.
